# Supplementary material for: Vardenafil Activity in Lung Fibrosis and In Vitro Synergy with Nintedanib
Source: Cells. 2021 Dec 11;10(12):3502. doi: 10.3390/cells10123502 (PMC8699915; doi:10.3390/cells10123502)
Supplement: Supplementary file 1 [file cells-10-03502-s001.zip › cells-1359697-supplementary.pdf]

Online Supplementary Materials for: Activity of Phosphodiesterase-5 Inhibitor Vardenafil in Lung Fibrosis and *In Vitro* Synergy with Nintedanib

SUPPLEMENTARY MATERIALS AND METHODS

Cell culture. Several murine and human cell lines were used in this study. IMR-90 cells were cultured in high-glucose DMEM (Life Technologies, Inc.) and AKR-2B cells were cultured in McCoy's 5A (modified) media (Gibco). Both were supplemented with 10% fetal bovine serum (FBS; HyClone™ GE Healthcare Life Sciences). Human IPF fibroblasts were cultured in FGM-2 with FGM-2 SingleQuot Kit Supplement & Growth Factors (LONZA). For TGF-β1 signaling experiments, IMR-90 cells were seeded at 2.0 x 10^5 in 6-well culture plates, grown to 80-90% confluence, and subsequently serum starved by replacing medium with 0.1% FBS/DMEM for 24 h. AKR-2B fibroblasts were seeded at 1.0 x 10^5, grown to 70-80% confluence, and subsequently serum starved by replacing medium with 0.1% FBS/McCoy's 5A for 24 h. Human IPF fibroblasts were seeded at 1.5 x 10^5, grown to 70-80% confluence, and subsequently serum starved by replacing medium with 0.1% FBS/FGM-2 for 24 h (FGM-2 with FGM-2 SingleQuot Kit Supplement & Growth Factors; LONZA).

Human IPF fibroblasts were purchased from LONZA. This cell line was obtained from an IPF patient as previously characterized [1, 2]. Cell line CCL-210, a normal primary lung fibroblast and all other cell lines (IMR-90, AKR-2B, RLE-6TN) were purchased from the American Type Culture Collection. TGF-β1 was obtained from R&D Systems.

Reagent Sources. PDE5 inhibitor vardenafil. PDE5 inhibitor vardenafil was obtained from either Sequoia Research Products Ltd. (vardenafil citrate), Sigma Aldrich, or Selleckchem (vardenafil HCl). Nintedanib (BIBF 1120; nintedanib) was obtained from Cayman Chemical. Antibodies used in this study were as follows: fibronectin (Sigma Aldrich, F3648), CTGF (Santa Cruz Biotechnology, sc-14939), p-SMAD3 (Cell Signaling 95205), total SMAD3 (Abcam AB 28379), and GAPDH (Millipore MAB374). Secondary antibodies used for fibronectin ELISA included Anti-Rabbit IgG peroxidase (Sigma Aldrich, A0545) and Anti-Rabbit IgG-HRP (SantaCruz Biotechnolog sc-2004).

Gene expression of human IPF and normal lung fibroblasts. To investigate whether PDE5 inhibitor targetable pathways were expressed in IPF fibroblastic lung cells (IPF-74), gene expression profiling was performed using the Affymetrix U133 plus 2 array, which provides approximately 47,000 transcripts from the human genome. Total RNA from the IPF-74 cells and the CCL-210 normal lung fibroblast cell lines was extracted using the Qiagen RNeasy Mini kit according to manufacturer's instructions.

Box plots were generated from the pre-processed data to assess the adequacy of normalization and chip quality. The overall distribution for the 6 samples (IPF-74 vs. CCL-210 cell lines, baseline transcription levels analyzed on three separate days) was very similar. Before analysis, the mean intensity was computed for each probe set and the lowest 40% were removed, leaving a total of 28,643 probe sets (genes) for final analysis. Based on this study design, a paired T-test was deemed the most appropriate, which takes the factor of culture day into consideration. The top 50 genes with p-value <0.05 and fold change $<$ 3 standard deviations up or down-regulated between IPF-74 and

CCL-210 are reported. Statistical analysis was performed by the Mayo Clinic Advanced Genomics Technology Center.

Effects of vardenafil on TGF-β1 driven collagen type I, alpha 1 (COL1A1), fibronectin, and thrombospondin 1 (TSP-1) expression by lung fibroblasts. Vardenafil was added to IMR-90 lung fibroblasts at the indicated concentration after overnight starvation for 30 min prior to the subsequent addition of TGF-β1 (5 ng/ml). The IMR-90 fibroblasts were incubated with the TGF-β1 containing media in the presence or absence of vardenafil at the indicated concentrations for an additional 24 h, and RNA was isolated from the cultures. Quantitative real-time RT-PCR (qPCR) was performed to determine the relative expression of COL1A1, fibronectin, and TSP-1 by the lung cells.

For qPCR analysis, the CFX96 Real-Time PCR detection System was utilized (Bio-Rad). RNA was extracted from cells using a Qiagen RNA isolation kit. To generate cDNA for qPCR analysis, SuperScript® III Reverse Transcriptase was employed (Life Technologies). To quantify the final cDNA PCR products, SYBR® Green PCR Master Mix (Life Technologies) was used. Conditions for the PCR reactions were as follows: 50°C for 2 min, 95°C for 2 min, followed by 40 cycles of 95°C for 15 sec, 60°C for 30 sec, and 72°C for 30 sec. During the 72°C temperature, analysis of the SYBR fluorophore for quantification was conducted. Relative expression levels of the respective transcripts were calculated by normalizing to the level of H3 histone mRNA by using comparative threshold cycle (ct) method, in which fold difference = 2-(Δct of target gene-Δct of reference). Primers for amplification of the respective cDNA was as

follows: Trombospondin-1 mRNA were 5'- CGACTGTGAAAAGATGGAGAATGC-3' and 5'- TGTTGTCAAGGGTGAGGAGGAC-3'. Collagen type I, alpha 1 (COL1A1) transcript was amplified with primers 5'-ACAGGGCGACAGAGGCATAAAG-3' and 5'-AACAGGACCAGCATCACCAGTG-3'. Fibronectin transcript was amplified with primers 5'-AAGAGGCAGGCTCAGCAAATG-3' and 5'-TTAGGACGCTCATAAGTGTCACCC-3'. For normalization to a housekeeping gene, H3 histone was used using the following primers: 5'-GGTAAAGCACCCAGGAAGCAAC-3' and 5'- ATTTCTCGCACCAGACGCTG-3'.

Activity of vardenafil in TGF-β1 induced morphologic transformation of fibroblasts *in vitro*. AKR-2B fibroblasts were cultured to confluence, serum starved overnight, and subsequently pretreated with 200 μM Vardenafil or DMSO control for 30 min. Cells were then left untreated (–) or stimulated (+) with 5 ng/ml TGF-β1 for 48 h. Photomicrographs were taken to identify morphologic differences.

Activity of vardenafil in TGF-β1 induced epithelial mesenchymal transformation of epithelial cells *in vitro*. In addition, studies were performed to determine whether vardenafil could suppress measures of EMT *in vitro*. To accomplish this, lung epithelial cells (RLE-6TN) cultured to confluence, and subsequently serum starved by replacing medium with serum-free DMEM for 24 h were used. The cells were then pretreated for 30 min with either 200 μM of vardenafil or negative control (DMSO) and subsequently left untreated or stimulated with 5 ng/mL TGF-β1 for up to 48 h. The levels of α-smooth muscle actin (α-SMA) expression under these conditions were

measured with real time quantitative PCR (Bio-Rad CFX96 q-PCR). Primers for detecting the α-SMA transcripts were 5'-CGATAGAACACGGCATCATCACC-3' and 5'- CAGAAGCATAGAGGG ACAGCACAG-3'.

Effect of vardenafil on anchorage independent growth. To further assess the effects of vardenafil in suppressing TGF-β1 fibroproliferative response, we measured the activity of vardenafil in suppressing anchorage independent growth of fibroblasts as previously reported [3]. To prevent cells from settling and adhering to the plate bottom, bottom plugs (1 mL) containing 0.8% Sea Plaque-agarose (FMC Corporation) and 10% FBS/DMEM were cast in 35-mm plates. AKR-2B fibroblasts ($1x10^4$ cells plate) in the presence or absence of 5 ng/mL TGF-β1 were seeded on top of these plugs, followed by layering on top plugs (1 ml) composed of 0.4% agarose, 10% FBS/DMEM. As indicated, in test chambers, the top plugs also contained 200 μM vardenafil. After 10 days of culture at 37°C, the number of colonies >25 μm in diameter were counted by microscopy using a 1.0-cm grid. Ten grid regions were counted on each of three plates. Quantification represents the average and SD of at least two independent experiments each performed in triplicate.

Effects of vardenafil on TGF-β1 signaling in fibroblasts. To assess SMAD activation, AKR-2B fibroblasts were treated as indicated for 45 minutes and the cultured cells were lysed with 50 mmol/L Tris-HCl (pH 7.4), 1% Triton X-100, 0.25% sodium deoxycholate, 150 mmol/L NaCl, 1 mmol/L EDTA, 1 mmol/L phenylmethylsulfonyl fluoride, 1 mmol/L Na_3VO_4, 5 mmol/L NaF, and 1X complete protease inhibitor (Roche

Applied Science). Equivalent total protein was separated by SDS-PAGE and the proteins transferred to nitrocellulose (Bio-Rad). The membranes were probed with a phospho-specific SMAD3 antibody or total SMAD3 antibody as listed above. Likewise, extracellular matrix proteins fibronectin and connective tissue growth factor (CTGF) were also analyzed at the doses noted. Specifically, fibroblasts were treated for 30 minutes with increasing doses of vardenafil, and then TGF-β1 added as described above. Additional TGF-b signaling assessments also included; serpin E1/PAI-1, αSMA, phospho-AKT, and phospho S6K (Thr389). Parallel blots were probed with GAPDH to verify equal protein loading.

Activity of vardenafil in TGF-β1 driven fibronectin production and morphological transformation of fibroblasts. We next examined whether vardenafil would suppress TGF-β1 driven morphological transformation *in vitro*. To accomplish this, AKR-2B fibroblasts were seeded at 1 x 10^5 in six-well tissue culture dishes, cultured to confluence, and subsequently serum starved by replacing medium with 0.1% FBS/McCoy's 5A for 24 h. The cells were then pretreated for 60 min with different concentrations of vardenafil and subsequently stimulated with 5 ng/mL TGF-β1 for 24 h. Multiple representative cell fields were examined and photographed.

Combination of vardenafil and nintedanib combination and fibroblast EMC production. *In vitro* studies first using AKR-2B cells and subsequently human IPF fibroblasts were further performed to investigate the effects of adding vardenafil to the approved anti-fibrotic agent nintedanib. In preliminary studies nintedanib had matrix

suppression activity in the cell culture system at concentrations <10 μM.

AKR-2B fibroblasts were seeded at 1 x 10^5 in six-well tissue culture dishes, cultured to 70-80% confluence, and subsequently serum starved by replacing medium with 0.1% FBS/McCoy's 5A for 24 h. Diseased human IPF fibroblasts were seeded at 1.5 x 10^5 in six-well tissue culture dishes, cultured to 70-80% confluence, and subsequently serum starved by replacing medium with 0.1% FBS/FGM-2 for 24 h. The cells were pretreated for 60 min with different doses of vardenafil or nintedanib or the combination dose and subsequently stimulated with 5 ng/mL TGF-β1 for 24 h after which the cells were harvested and lysed with cell lysis buffer (high detergent RIPA buffer, EDTA-free protease inhibitor, PMSF, NaOrtho, and NaF). After BCA protein assay, protein-normalized cell lysates and a fibronectin standard were plated on a 96 well plate. Fibronectin ELISA was performed using the aforementioned antibodies and developed using TMB substrate solution (3,3',5,5'-tetramethylbenzidine) and stopped with H_2SO_4 Fibronectin concentration was quantified spectrophotometrically.

Cell Proliferation and Viability Assay. Colorimetric XTT-based assays (Cell Proliferation Kit II (XTT)(Roche)) were then performed to quantify cell viability in the setting of drug administration and TGF-β1 stimulation to evaluate for potential drug-induced toxicity in AKR-2B fibroblasts and human IPF fibroblast lines treated with vardenafil and nintedanib. Fibroblast seeding and growth to appropriate confluence with subsequent serum starvation, drug inoculation, and TGF-β1 stimulation were performed per usual protocol. Twenty-four hours after drug inoculation, XTT labeling reagent (sodium 3´-[1-(phenylaminocarbonyl)- 3,4-tetrazolium]-bis (4-methoxy-6-nitro) benzene

sulfonic acid hydrate) and electron coupling reagent (N-methyldibenzopyrazine methyl sulfate) were added to each well and incubated for assay development over 4 to 6 hours. Orange formazan solution, directly correlating to mitochondrial dehydrogenase activity in sample cells, was spectrophotometrically quantified as a surrogate for cell viability and displayed as relative units.

Activity of vardenafil in bleomycin treated mouse model. Eight week old, female C57 black mice (~20 gram; Jackson Laboratories, Bar Harbor, ME) received intratracheal bleomycin (0.1 U diluted in 75 µl normal saline) using an aerosolizing needle (Penn-Century, Wyndmoor, PA, USA) while receiving ketamine/xylazine anesthesia. Vardenafil was prepared by solubilizing in 0.9% normal saline. The mice received intraperitoneal vardenafil daily at the indicated doses beginning on the day of bleomycin administration. The mice were examined daily for respiratory distress, weight loss, and general well-being. On day 19, the mice were euthanized by injection of pentobarbital (100 mg/kg), and the thorax opened. The lungs were then dissected free of the thoracic structures, and samples submitted for collagen analyses. Portion of excised lung were snap frozen in liquid nitrogen and nucleic acids extracted. Collagen type 1, alpha 1 (COL1A1) steady state mRNA expression was quantified by qPCR using mouse specific primers as follows 5'-GCAAGAATGGAGATGATGGG-3' and 5'-TCCGTTTTCACCAGGACTGC-3'.

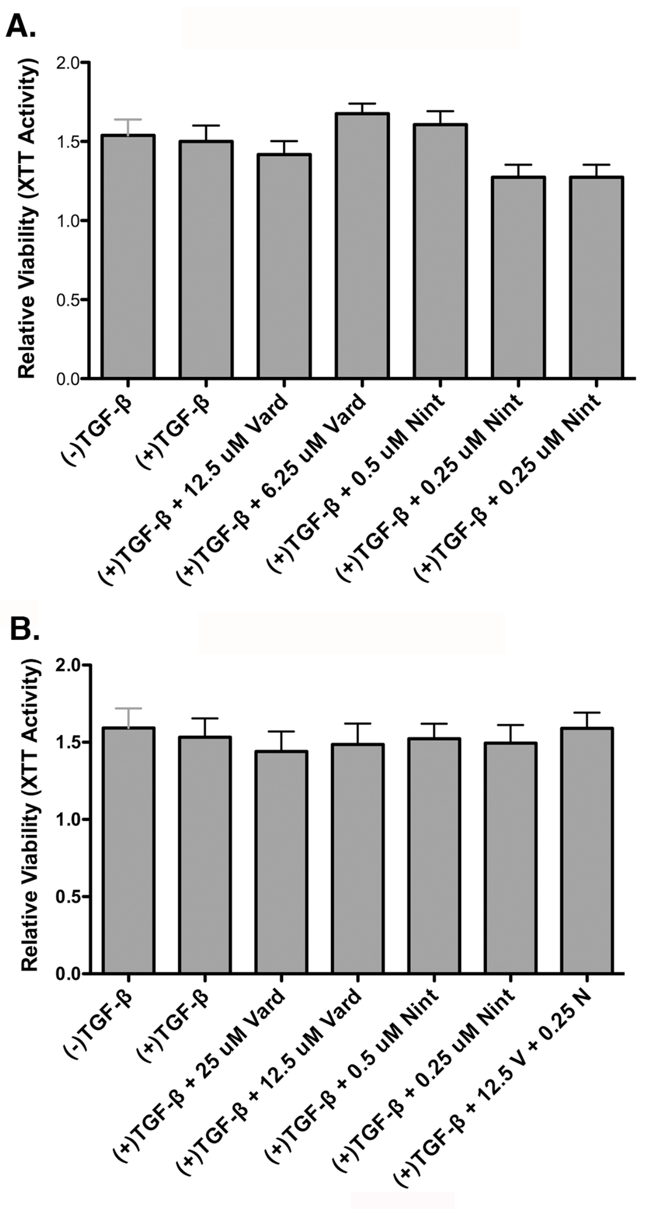

Supplementary Figure S1. XTT viability assays demonstrate no significant toxicity related to vardenafil or nintedanib, alone or combined A. AKR-2B fibroblasts were incubated with the test agents as indicated overnight and assessed for viability by XTT assay. All demonstrated preserved viability, defined as greater than 80% of controls. **B.** IPF fibroblasts were incubated with the test agents as indicated overnight and assessed for viability by XTT assay. All demonstrated preserved viability, defined as greater than 80% of controls.

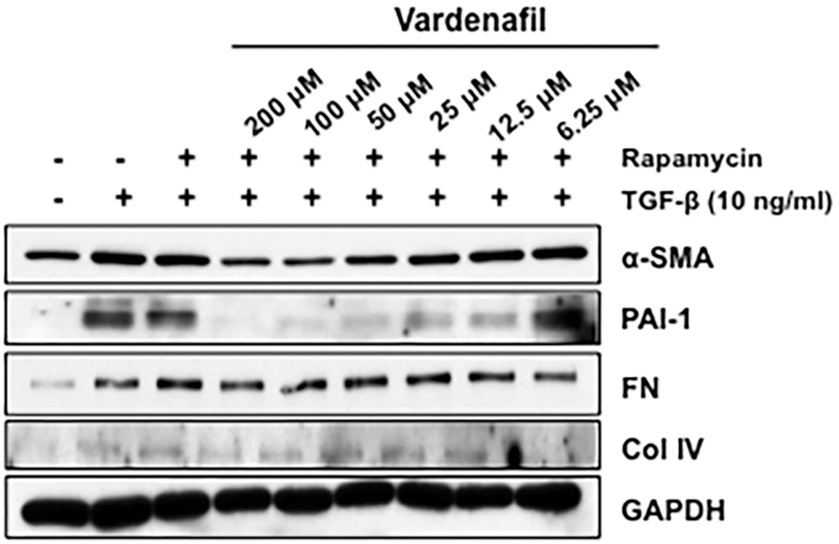

Supplementary Figure S2. mTOR1 activity is not necessary for the anti-fibrotic effect of vardenafil. Quiescent fibroblasts (AKR-2B) in 0.1% FBS/DMEM were pretreated for 60 min with Rapamycin (mTORC1 inhibitor; 100 nM). Vehicle (-) or TGF-β (+) was directly added to a final concentration of 10 ng/ml and following 24 h incubation, lysates were prepared and Western blotted for α-SMA, PAI-1, FN and Col IV. GAPDH was used as a loading control. Data are representative of 3 separate experiments. These data indicate that the anti-fibrotic effect of vardenafil does not depend on mTORC1 activity as shown by profibrotic marker expression.

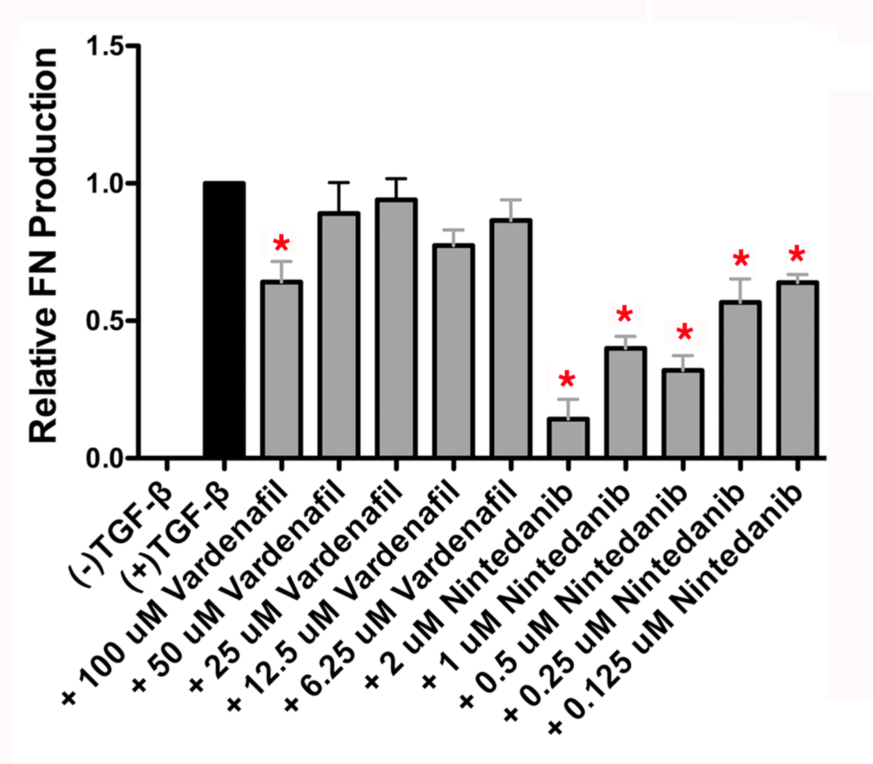

Supplementary Figure S3. Vardenafil inhibits TGF-β1-mediated fibronectin ECM production in AKR-2B fibroblasts. Fibronectin fibronectin production was assesed by ELISA in AKR-2B fibroblasts treated with TGF-b1 and vardenafil or nintedanib at the indicated doses. Data from six experiments with results expressed mean ± SEMs. (*Denotes $P<0.05$ compared to maximal TGF-β1 induced expression of fibronectin, defined as 1.0 relative units)

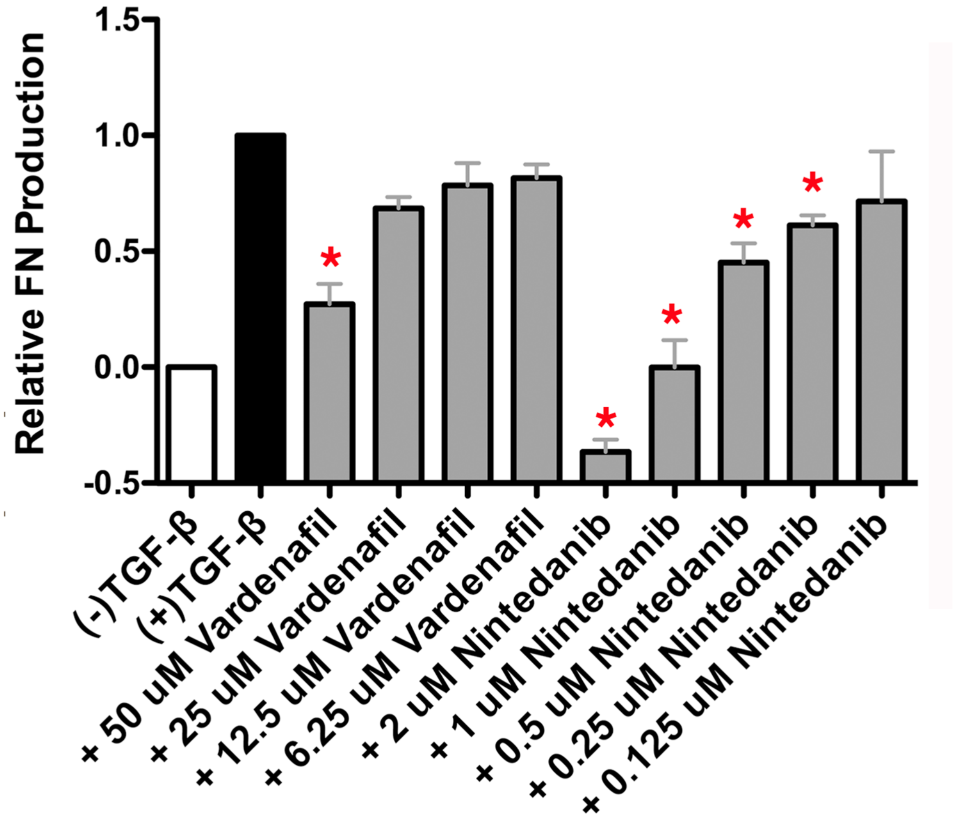

Supplementary Figure S4. Vardenafil inhibits TGF-β1-mediated fibronectin ECM production in human IPF fibroblasts. Fibronectin fibronectin production was assesed by ELISA in IPF fibroblasts treated with TGF-β1 and vardenafil or nintedanib at the indicated doses. Data from nine experiments with results expressed as mean ± SEM (*Denotes P<0.05 compared to maximal TGF-β1 induced expression of fibronectin, defined as 1.0 relative units)

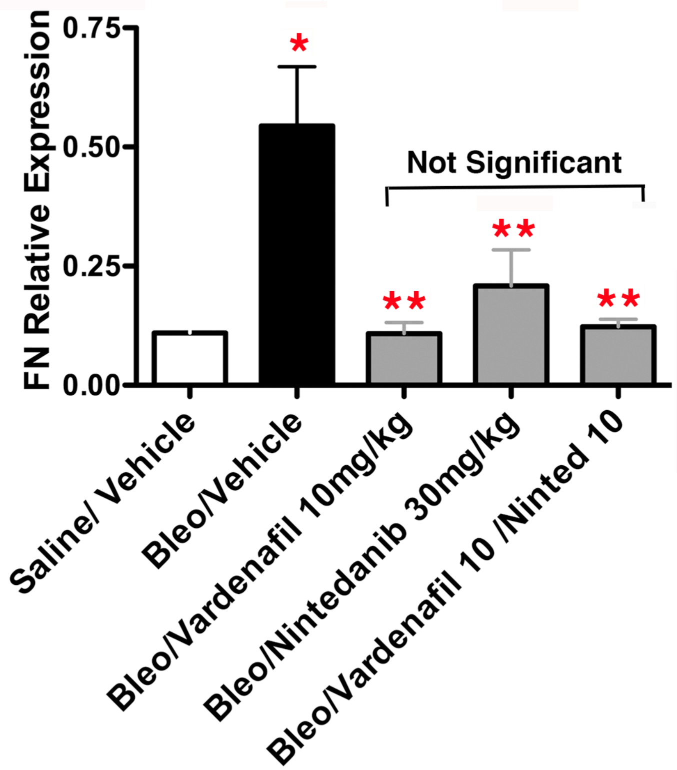

Supplemantary Figure S5. Vardenafil does not demonstrate additional synergistic suppression of *in vivo* extracellular matrix fibronectin generation in bleomycin treated mice. Mice received intratracheal bleomycin and were treated with vardenafil over 19 days as detailed under methods. The size of each group at the time of injury was nine. Fibrosis in the lungs was measured by the generation of extraceellar matrix fibronectin-EDA in the various groups. Bleomycin induced significant new fibronectin generation (* denotes $P<0.05$ compared to saline/vehicle control), and both vardenafil and nintedanib significantly decreased the generation of extracellular matrix fibronectin-EDA (** denotes $P<0.05$ compared to bleomyicn/vehicle control). However, the combination of the two agents did not demonstrate any additional significant benefit over either agent alone ($P>0.05$, Not Signficant).

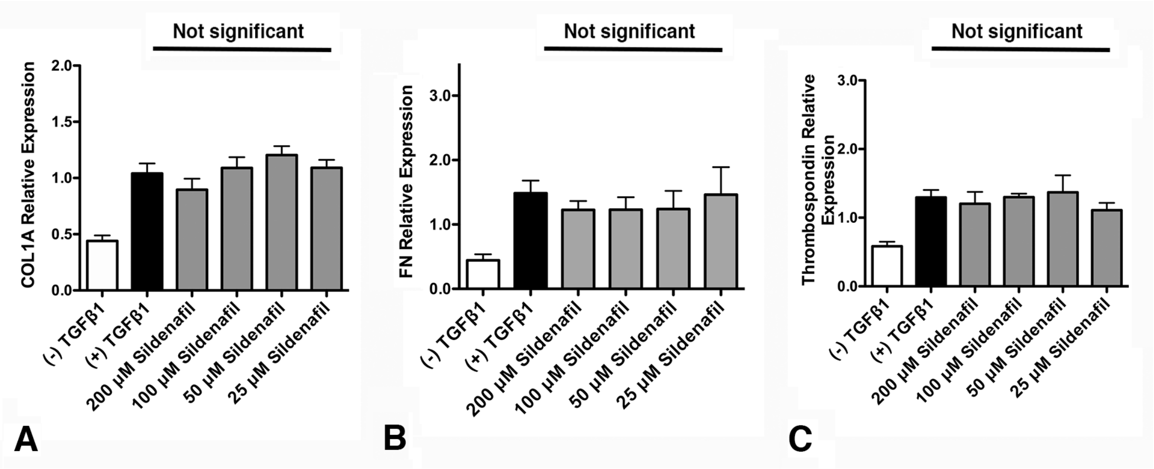

Supplementary Figure S6. Sildenafil does not significantly inhibit TGF-β1-mediated matrix-associated mRNA levels in lung fibroblasts. Effect of the respective mRNA transcripts in human IMR-90 fibroblasts stimulated with TGF-β1 after 24 h in the presence of the indicated vardenafil concentrations. **A.** Collagen type I, alpha 1 (COL1A1), **B.** Fibronectin, and **C.** Thrombospondin (TSP-1) relative mRNA expression in IMR-90 lung fibroblasts. Data are means ± SEM. In contrast to our observations with vardenafil (Figure 1), there were no significant suppressions of matrix components comparing tested condition to control maximal stimulation in the presence TGF-β1 but in the absence of sildenafil.

Supplementary References

1. Larsson O, Diebold D, Fan D, Peterson M, Nho RS, Bitterman PB, Henke CA: **Fibrotic myofibroblasts manifest genome-wide derangements of translational control**. *PLoS One* 2008, **3**(9):e3220.
2. Khalil W, Xia H, Bodempudi V, Kahm J, Hergert P, Smith K, Peterson M, Parker M, Herrera J, Bitterman PB *et al*: **Pathologic Regulation of Collagen I by an Aberrant PP2A/HDAC4/miR-29 Signal Axis in IPF Fibroblasts**. *Am J Respir Cell Mol Biol* 2015.
3. Daniels CE, Wilkes MC, Edens M, Kottom TJ, Murphy SJ, Limper AH, Leof EB: **Imatinib mesylate inhibits the profibrogenic activity of TGF-beta and prevents bleomycin-mediated lung fibrosis**. *J Clin Invest* 2004, **114**(9):1308-1316.
